# Preserved Skeletal Muscle Mitochondrial Function, Redox State, Inflammation and Mass in Obese Mice with Chronic Heart Failure

**DOI:** 10.3390/nu12113393

**Published:** 2020-11-04

**Authors:** Gianluca Gortan Cappellari, Aneta Aleksova, Matteo Dal Ferro, Antonio Cannatà, Annamaria Semolic, Michela Zanetti, Jochen Springer, Stefan D. Anker, Mauro Giacca, Gianfranco Sinagra, Rocco Barazzoni

**Affiliations:** 1 Department of Medical, Surgical and Health Sciences—University of Trieste, 34149 Trieste, Italy; gigici2@iol.it (G.G.C.); aaleksova@gmail.com (A.A.); semolic@units.it (A.S.); zanetti@units.it (M.Z.); giacca@icgeb.org (M.G.); 2 Cardiothoracovascular Department, Azienda Sanitaria Universitaria Giuliano Isontina (ASUGI), 34149 Trieste, Italy; dalferro@icgeb.org (M.D.F.); anto.cannata@gmail.com (A.C.); gianfranco.sinagra@asugi.sanita.fvg.it (G.S.); 3 Molecular Medicine Laboratory, International Centre for Genetic, Engineering and Biotechnology (ICGEB), 34149 Trieste, Italy; 4 Berlin-Brandenburger Centrum für Regenerative Therapien, Charité—Universitätsmedizin Berlin, 13353 Berlin, Germany; jochen.springer@charite.de (J.S.); s.anker@cachexia.de (S.D.A.); 5 School of Cardiovascular Medicine & Sciences, King’s College London, London SE5 9NU, UK

**Keywords:** chronic heart failure, skeletal muscle, sarcopenia, obesity, obesity paradox

## Abstract

**Background**: Skeletal muscle (SM) mitochondrial dysfunction, oxidative stress, inflammation and muscle mass loss may worsen prognosis in chronic heart failure (CHF). Diet-induced obesity may also cause SM mitochondrial dysfunction as well as oxidative stress and inflammation, but obesity per se may be paradoxically associated with high SM mass and mitochondrial adenosine triphosphate (ATP) production, as well as with enhanced survival in CHF. **Methods**: We investigated interactions between myocardial infarction(MI)-induced CHF and diet-induced obesity (12-wk 60% vs. standard 10% fat) in modulating gastrocnemius muscle (GM) mitochondrial ATP and tissue superoxide generation, oxidized glutathione (GSSG), cytokines and insulin signalling activation in 10-wk-old mice in the following groups: lean sham-operated, lean CHF (LCHF), obese CHF (ObCHF; all *n* = 8). The metabolic impact of obesity per se was investigated by pair-feeding ObCHF to standard diet with stabilized excess body weight until sacrifice at wk 8 post-MI. **Results**: Compared to sham, LCHF had low GM mass, paralleled by low mitochondrial ATP production and high mitochondrial reative oxygen species (ROS) production, pro-oxidative redox state, pro-inflammatory cytokine changes and low insulin signaling (*p* < 0.05). In contrast, excess body weight in pair-fed ObCHF was associated with high GM mass, preserved mitochondrial ATP and mitochondrial ROS production, unaltered redox state, tissue cytokines and insulin signaling (*p* = non significant vs. Sham, *p* < 0.05 vs. LCHF) despite higher superoxide generation from non-mitochondrial sources. **Conclusions**: CHF disrupts skeletal muscle mitochondrial function in lean rodents with low ATP and high mitochondrial ROS production, associated with tissue pro-inflammatory cytokine profile, low insulin signaling and muscle mass loss. Following CHF onset, obesity per se is associated with high skeletal muscle mass and preserved tissue ATP production, mitochondrial ROS production, redox state, cytokines and insulin signaling. These paradoxical and potentially favorable obesity-associated metabolic patterns could contribute to reported obesity-induced survival advantage in CHF.

## 1. Introduction

Chronic heart failure (CHF) is a major cause of morbidity and mortality in the general population, with increasing prevalence worldwide [1,2,3]. Besides the severity of myocardial dysfunction, CHF patients’ outcome is strongly and negatively influenced by loss of skeletal muscle mass and function [4,5,6,7,8,9], and a pattern of metabolic abnormalities affecting tissue energy metabolism and anabolic responses is emerging as a relevant contributor to skeletal muscle derangements [10,11,12,13,14,15,16]. In particular, metabolic changes may reportedly include mitochondrial dysfunction, altered redox state, inflammation and insulin resistance [12,17,18,19,20,21,22,23,24]. Some although not all reports have further suggested that metabolic alterations, particularly those involving mitochondrial function and redox state, are more pronounced in skeletal muscle in CHF patients with reduced compared to preserved ejection fraction [25]. Indeed, we recently showed that myocardial infarction-induced CHF with reduced ejection fraction leads to low mitochondrial oxidative enzyme activities, associated with pro-inflammatory transcription factors activation and low insulin signaling [26] that might directly impair skeletal muscle performance and anabolism [13]. Mitochondrial changes could also contribute to enhance reactive oxygen species generation [12,27,28], which might per se further enhance inflammation, insulin resistance, further mitochondrial dysfunction, excitation-contraction uncoupling and ultimately muscle catabolism [20,27,29,30,31,32]. 

Mechanisms underlying CHF-induced skeletal muscle metabolic abnormalities and catabolism remain only partly explored while, based on the above considerations their understanding is a major research and clinical priority. Nutritional status is a potential major modulator of skeletal muscle metabolism, but little is known on its muscle metabolic impact in CHF. In recent years obesity has been paradoxically associated with better outcomes and enhanced survival in CHF, but potential interactions between obesity and CHF to differentially modulate skeletal muscle metabolic responses remain unknown [4,13,33,34,35]. Although its long-term metabolic complications include skeletal muscle oxidative stress, inflammation and insulin resistance [28,36,37], fat-induced enhancement of mitochondrial biogenesis and high muscle mitochondrial oxidation capacity have been reported in experimental and clinical obesity models [38,39]. Intriguingly, no changes or even increases in insulin signaling activation may also characterize early obesity stages in a time-dependent fashion [40,41,42]. In addition and also importantly, epidemiological studies have reported enhanced skeletal muscle mass in obese individuals to parallel increments of total body mass [43]. In the current study we therefore investigated interactions between myocardial infarction-induced CHF with reduced ejection fraction and high-fat diet-induced obesity (60% vs. standard 10% dietary calories as fat) in modulating mitochondrial adenosine triphosphate (ATP) and reactive oxygen species (ROS) generation at various cellular levels, tissue redox state markers, pro- and anti-inflammatory cytokines and insulin signaling protein activation. To this aim, myocardial infarction was induced by coronary artery ligation in 10-wk-old lean or high-fat diet-induced obese mice. In order to investigate the role of obesity per se and to prevent potential confounding effects of simultaneous high-fat feeding (High-Fat-Metabolism), obese animals were pair-fed to the same standard diet and caloric intake of lean animals after surgery and until sacrifice. 

## 2. Methods 

### 2.1. Study Protocol

Thirty10-week-old male CD1 mice (Harlan, San Piero al Natisone, Italy) mice were randomly assigned to the following diets which were administered ad libitum: standard (Harlan 2018, Fat = 9% total calories, L, *n* = 20) or high-fat diet (Harlan TD.6414, Fat = 60% total calories, Ob, *n* = 10). Mice were kept at constant temperature and humidity and exposed to 12 h light/dark cycles in the International Centre for Genetic Engineering and Biotechnology (ICGEB, Trieste, Italy) animal facility. The study was conducted in compliance with national and international laws and policies. Study design and procedures were approved by the ICGEB Animal Welfare Board and Ethical Committee and by the National Animal Experimentation Authority (6442015PR). After 12 weeks, heart failure was induced in 8 animals from each diet by permanent left anterior descending (LAD) coronary artery ligation (*n* = 11 each group, CHF) as previously described [44,45]. Briefly, after anesthesia was induced with ketamine (100 mg kg^−1^) and xylazine (10 mg kg^−1^), mice were placed on a warmed pad to maintain body temperature, an endothracheal tube was placed and were mechanically ventilated (Model 131 rodent ventilator, Nemi Scientific Inc., Medway, MA, USA). Following left thoracotomy, pericardiotomy and heart exposure, LAD descendent branch was ligated with an 8-0 silk suture under stereomicroscope (Leica) visualization. Following MI confirmation by observation of left ventricle’s anterior wall whitening, the chest was closed and mice allowed to recover spontaneous breathing. The other animals were treated with sham operation (S). To further evaluate cardiac function and confirm CHF, 2-D transthoracic echocardiography was performed using a Vevo 770 Ultrasound (Visualsonics, Bothell, WA, USA) device with a 30-MHz linear array transducer, both after surgery and one week before sacrifice. Animals who underwent LAD ligature but whose left ventricular ejection fraction (LVEF) was not reduced to <50% were excluded from the study, leaving *n* = 8 animals/group. As expected, all sham operated animals had LVEF >50%. Following operation, all mice were pair-fed with standard diet to assess the impact of obesity per se on investigated parameters. Mice were sacrificed at week 20, three hours after food was removed, following anesthesia induction [27]. Left gastrocnemius muscle was then surgically isolated, explanted, cleansed and weighed. Blood collected by heart puncture in EDTA vials and placed in ice until separation by centrifugation. Epididimal fat pads and retroperitoneal fat was isolated by careful microdissection and weighted. Tissue samples were the placed in iced saline solution for immediate transfer to the laboratory performing ex-vivo assessments, or snap-frozen in liquid nitrogen for further analyses [28].

### 2.2. Blood Glucose 

Plasma glucose was determined by standard enzymatic-colorimetric assay [29].

### 2.3. Mitochondrial Function

ATP synthesis rate in isolated mitochondria was measured ex-vivo in freshly isolated mitochondria by luciferin-luciferase luminescence assay in a microplate luminometer (Synergy 2 SL, BioTek, Winooski, VT, USA) as previously described [26,28]. Several combinations of respiratory substrates were contemporarily tested using the following final reaction concentrations (mmol/L): 0.25 pyruvate, 0.0125 palmitoyl-L-carnitine, 2.5 α-ketoglutarate, 0.25 malate (PPKM); 0.025 palmitoyl-L-carnitine, 0.5 malate (PCM); 20 succinate, 0.1 rotenone (SR); 10 glutamate, 5 malate (GM). Measurements obtained were normalized by sample citrate synthase activity, measured spectrophotometrically as referenced [28].

### 2.4. Superoxide Generation and Redox State

Superoxide anion generation in gastrocnemius lateralis was measured ex-vivo in tissue homogenate using the lucigenin chemiluminescent method as referenced [27,46,47]. Differences between groups in superoxide production from specific sources were assessed by measuring the impact of the addition of a source-specific inhibitor on superoxide-related light emission from tissue homogenate incubated with a source-specific substrate. In detail the following inhibitor/substrate combinations were used for each of the following superoxide generation sources: uncoupling agent Carbonyl cyanide m-chlorophenyl hydrazone (5 µmol/L)/respiratory substrate succinate (10 mmol/L) for mitochondria; 200 µmol/L Diphenylhydantoin (DPI)/1 mmol/L Nicotinamide adenine dinucleotide phosphate (NADPH) for NADPH-oxidase and 200 µmol/L Oxypurinol/500 µmol/L Xanthine for xanthine oxidase. Sample protein content (Bicinchoninic acid assay, Pierce, Rockford, IL, USA) was used to normalize results prior to comparison. 

Tissue total and oxidized glutathione were assessed spectrophotometrically on ~50 mg of gastrocnemius muscle homogenized in 5% (wt/vol.; +4 °C) metaphosphoric acid, using the 5,5′-dithiobis-(2-nitrobenzoic acid) (DTNB) conversion rate method as previously described [28,48]. To measure the oxidated fraction (GSSG), samples were previously incubated with 2-vynilpiridin 10% (*v*/*v*) and subsequently neutralized by addition of triethanolamine 16.6% (*v*/*v*) [27]. 

### 2.5. Protein Expression and Phosphorylation

Tissue cytokine levels and insulin signaling and protein anabolism signaling activation were measured using high-throughput xMAP magnetic beads technology with a Magpix reader (Luminex, Austin, TX, USA). Commercial kits used to determine both cytokine profile and insulin signaling protein expression and phosphorylation at Insulin receptor substrate 1 (IRS-1^S312^), Protein kinase B (AKT^S473^), Glycogen synthase kinase 3β (GSK-3β^S9^), mammalian target of rapamycin (mTOR^S2448^), Proline-rich AKT substrate protein (PRAS40^T246^) and Ribosomal protein S6 kinase (P70S6K^T421/S424^) were purchased form Millipore (Billerica, MA, USA) and used according to manufacturer’s instructions as previously published [27,28,36]. Data were analyzed using Milliplex Analyst software (Millipore, Billerica, MA, USA). Phosphorylation of insulin signaling pathway effectors is expressed as a phospho-protein/expression of the same protein ratio. Cytokine levels are normalized by homogenate protein content.

### 2.6. Statistical Analysis

Groups were compared using a Student t-test for unpaired or paired data or One-Way ANOVA for unpaired data followed by a Tukey post-hoc test, as appropriate. *p* values < 0.05 were considered statistically significant. Analysis was performed using GraphPad Prism v.5, GraphPad Software, San Diego, CA, USA.

## 3. Results

### 3.1. Calorie Intake, Body and Tissue Weight and Plasma Glucose 

Calorie intake: before MI and CHF induction, calorie intake was comparable by design in the two groups receiving control diet, with markedly higher intake in the high-fat diet group. After MI and CHF induction, ObCHF were switched to control diet by design, resulting in comparable caloric intake to Sham and LCHF (*p* = NS; Table 1). 

Body weight: following dietary interventions and before MI induction, body weight was comparable in groups receiving control diet while it was expectedly markedly higher in high-fat diet-fed animals. Final body weight at sacrifice was lower in LCHF than in Sham. In ObCHF, body weight remained higher than in both Sham and LCHF groups, but it did not increase compared to weight before MI and CHF induction.

**Figure 1:**
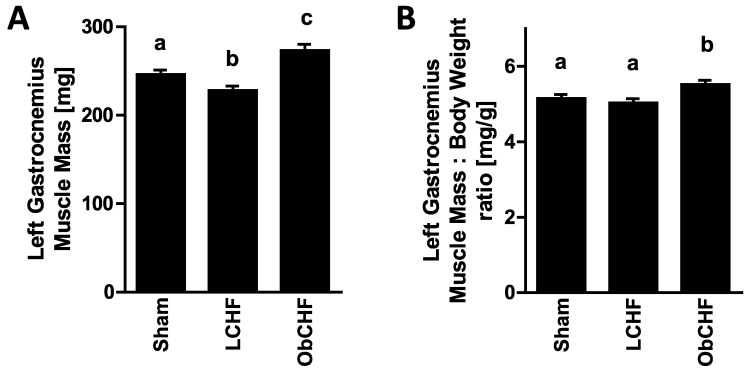
Muscle mass. Impact of previous 12-weeks diet-induced obesity in a mouse model after myocardial infarction-induced chronic heart failure and 8-week pair-feeding (ObCHF) on left gastrocnemius muscle mass compared to previously lean (LCHF) and to lean sham operated animals (Sham) in absolute (**A**) or relative to body weight (**B**) values. *p* < 0.05 among groups not sharing a letter, mean ± standard error of mean (SEM), *n* = 8/group.

Tissue weight: Compared to the Sham group, gastrocnemius muscle (GM) weight was lower in LCHF whereas it was highest in ObCHF (Figure 1A). GM weight was also found to be higher in ObCHF when normalized by total body weight (Figure 1B). Compared to Sham, left retroperitoneal and epididimal fat pads were comparable or lower in LCHF. In contrast, they were comparable or higher in ObCHF (Table 1).

Plasma glucose: plasma glucose was comparable in the Sham and LCHF groups while it was higher in ObCHF.

### 3.2. Skeletal Muscle Mitochondrial ATP and ROS Production 

**Figure 2:**
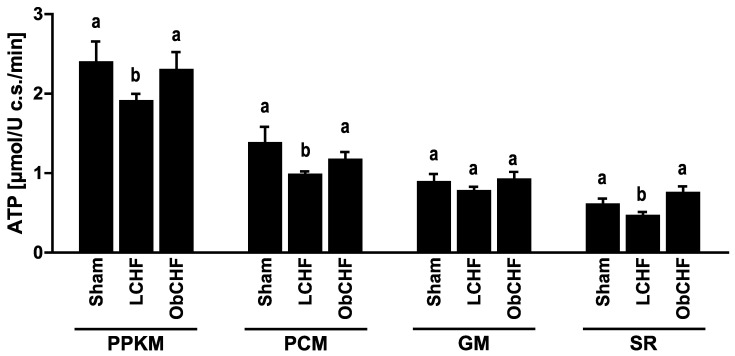
Muscle adenosine triphosphate (ATP) synthesis rate. Impact of previous 12-weeks diet-induced obesity in a mouse model after myocardial infarction-induced chronic heart failure and 8-week pair-feeding (ObCHF) on left gastrocnemius ATP synthesis rate in intact isolated mitochondria with different respiratory substrates (PPKM: Pyruvate+Palmitoyl-L-Carnintine+α-Ketoglutarate+Malate; PCM: Palmitoyl-L-Carnintine+Malate; GM: Glutamate+Malate; SR: Succinate+Rotenone) compared to previously lean (LCHF) and to lean sham operated animals (Sham). *p* < 0.05 among groups not sharing a letter, mean ± standard error of mean (SEM), *n* = 8/group.

**Figure 3:**
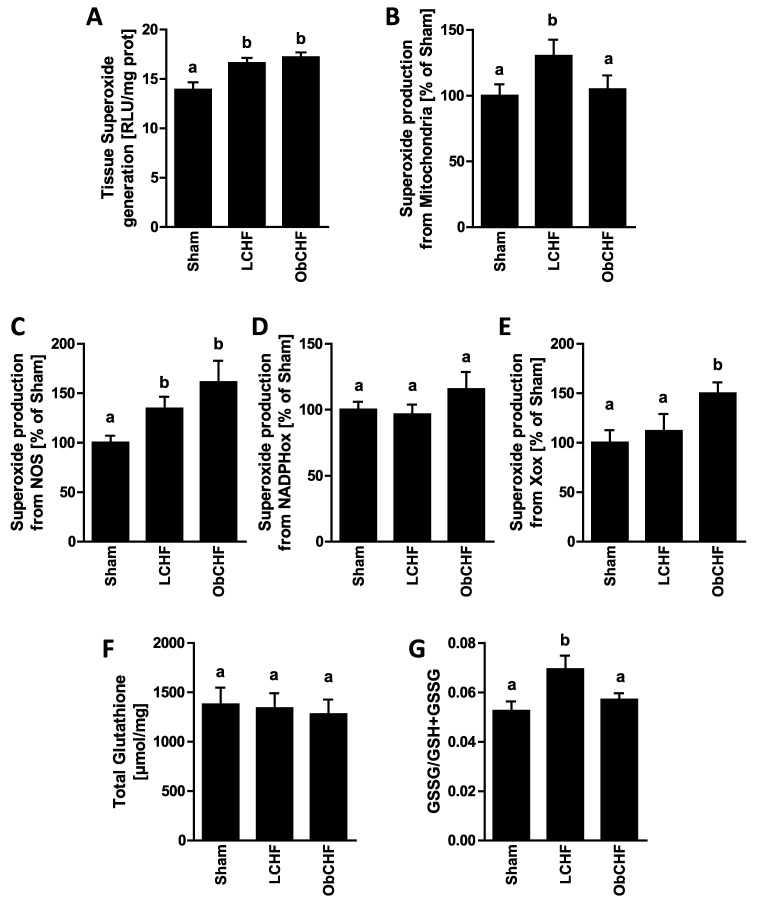
Muscle superoxide generation and redox state. Impact of previous 12-weeks diet-induced obesity in a mouse model after myocardial infarction-induced chronic heart failure and 8-week pair-feeding (ObCHF) on left gastrocnemius (**A**) total tissue, (**B**) mitochondria-, (**C**) uncoupled nitric oxide synthase (NOS)-, (**D**) Nicotinamide adenine dinucleotide phosphate oxidase (NADPHox)- and (**E**) Xanthine oxydase (Xox)-related superoxide generation as well as on (**F**) total and (**G**) oxydated (GSSG) over total (GSSG+GSH) tissue glutathione compared to previously lean (LCHF) and to lean sham operated animals (Sham). *p* < 0.05 among groups not sharing a letter, mean ± standard error of mean (SEM), *n* = 8/group.

Compared to Sham, LCHF animals had lower GM mitochondrial ATP production capacity (Figure 2) and high tissue total and mitochondrial superoxide production (Figure 3). In contrast, skeletal muscle ATP production was preserved in ObCHF (Figure 2) with mitochondrial superoxide comparable to the Sham group. High total GM superoxide production in ObCHF was conversely entirely attributable to non-mitochondrial sources including Nitric Oxide Synthase (NOS) and Xanthine Oxidase (Figure 3). GM tissue redox state as reflected by oxidized over total glutathione (GSSG/GSH+GSSG) was more pro-oxidative in LCHF and comparable in Sham and ObCHF (Figure 3).

### 3.3. Skeletal Muscle Cytokine Profile 

**Figure 4:**
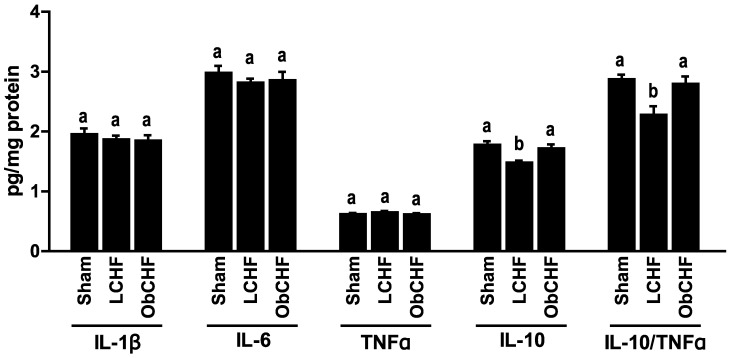
Muscle cytokine profile. Impact of previous 12-weeks diet-induced obesity in a mouse model after myocardial infarction-induced chronic heart failure and 8-week pair-feeding (ObCHF) on left gastrocnemius tissue protein levels of pro-inflammatory Interelukin (IL) 1β and 6 and Tumor Necrosis Factor α (TNFα), anti-inflammatory IL-10 and anti-inflammation index IL-10/TNFα ratio compared to previously lean (LCHF) and to lean sham operated animals (Sham). *p* < 0.05 among groups not sharing a letter, mean ± standard error of mean (SEM), *n* = 8/group.

Gastrocnemius muscle cytokine patterns paralleled those observed in mitochondrial superoxide emission. In particular, despite absolute levels of tested pro-inflammatory cytokines were comparable among groups, LCHF had selectively low anti-inflammatory IL-10 with unbalanced ratios to pro-inflammatory cytokines (IL-10/TNFα) compared to Sham. In contrast, ObCHF had preserved cytokine patterns compared to Sham animals (Figure 4). 

### 3.4. Skeletal Muscle Insulin Signalling

**Figure 5:**
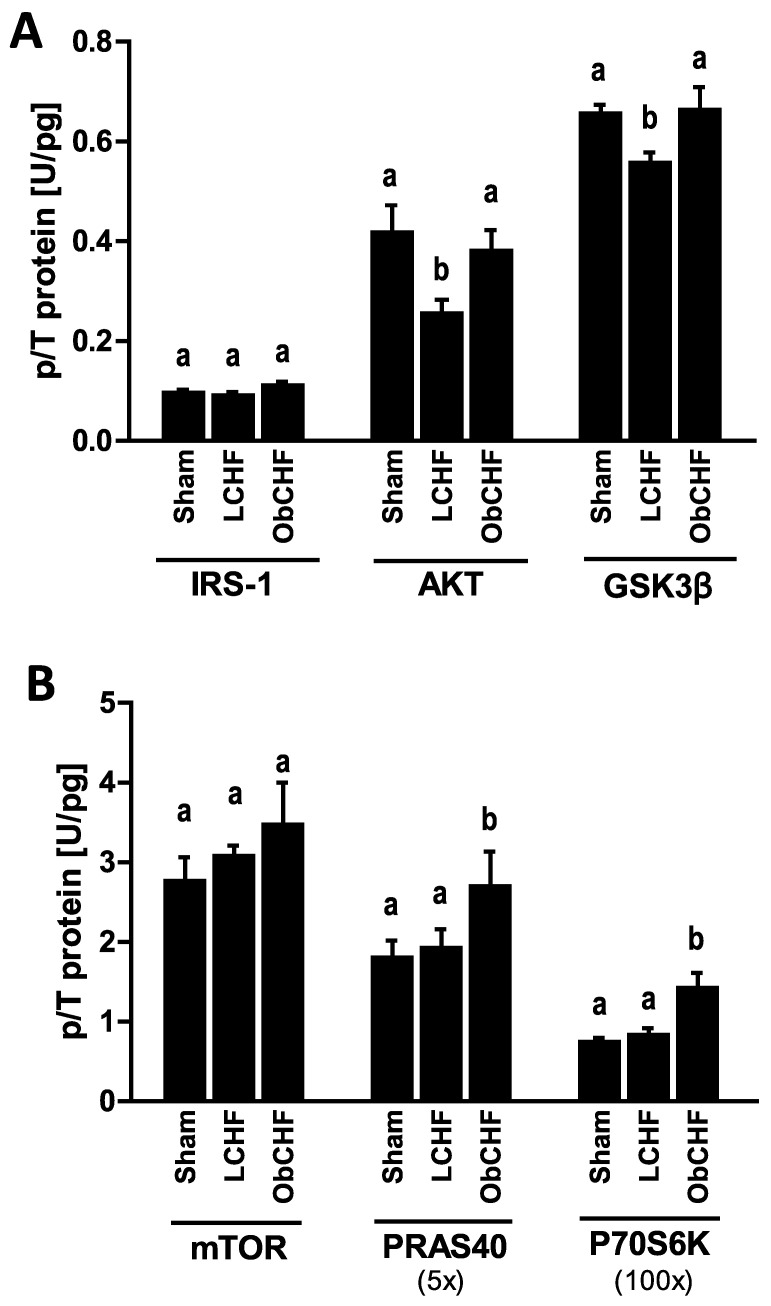
Muscle insulin and protein anabolism signaling activation. Impact of previous 12-weeks diet-induced obesity in a mouse model after myocardial infarction-induced chronic heart failure and 8-week pair-feeding (ObCHF) on left gastrocnemius activation in terms of relative phosphorylation (p/T) of (**A**) Insulin receptor substrate 1 (IRS-1^S312^), Protein kinase B (AKT^S473^), Glycogen synthase kinase 3β (GSK-3β^S9^) and (**B**) mammalian target of rapamycin (mTOR^S2448^), Proline-rich AKT substrate protein (PRAS40^T246^) and Ribosomal protein S6 kinase (P70S6K^T421/S424^) to total protein ratio compared to previously lean (LCHF) and to lean sham operated animals (Sham). *p* < 0.05 among groups not sharing a letter, mean ± standard error of mean (SEM), *n* = 8/group.

Gastrocnemius muscle activating phosphorylations of insulin signaling protein also reflected opposite changes in mitochondrial superoxide production and pro-inflammatory cytokine patterns. In particular, compared to Sham LCHF had selectively low phosphorylated AKT and GSK-3β with similar PRAS40 and P70S6K, while ObCHF had preserved phosphorylated AKT and GSK-3β and high phosphorylated PRAS40 and P70S6K (Figure 5). Total protein levels did not differ among study groups in the current model (data not shown).

## 4. Discussion

The current study demonstrated that in a MI-induced rodent model of CHF with reduced ejection fraction: (1) CHF leads to low gastrocnemius skeletal muscle mass with derangements of muscle metabolic pathways regulating tissue energy metabolism and mass maintenance; the latter include impaired mitochondrial ATP production, higher superoxide generation selectively at mitochondrial level with pro-oxidative redox state, pro-inflammatory changes in cytokine profile and lower insulin signaling; (2) obesity per se does not worsen CHF-induced metabolic derangements, but rather appears to play a paradoxically protective role with higher gastrocnemius muscle mass and mitochondrial ATP production, as well as preserved mitochondrial superoxide generation, tissue redox state, cytokine profile and insulin signaling. Our findings therefore identify paradoxical and potentially favorable obesity-associated skeletal muscle metabolic patterns, that likely contribute to preserve skeletal muscle mass and may thereby provide a novel metabolic basis for the obesity-induced survival advantage reported in CHF.

The study provides a comprehensive investigation of previously-reported metabolic derangements that potentially cluster to contribute to loss of skeletal muscle mass in CHF. In lean animals, we clearly show that CHF has a negative impact on energy metabolism with mitochondrial dysfunction both in terms of ATP and superoxide production, thereby likely directly contributing to altered redox state, altered cytokine profile and lower insulin signaling activation. The negative role of obesity as a risk factor for chronic heart failure is well established [49,50,51], but in recent years, a more complex relationship has emerged and a survival advantage, defined as obesity paradox, has been reported for obese individuals in CHF as well as other chronic diseases [33,34,49,52]. The current results provide new insight on potential mechanisms underlying the obesity paradox, by demonstrating that obesity per se may prevent derangements in skeletal muscle energy metabolism and tissue-catabolic pathways that characterize CHF-induced loss of skeletal muscle mass and ultimate worsening of patient outcomes and survival. 

The current findings in obese CHF animals, while novel and partly unexpected, are notably in agreement with previous reports that obesity and may not impair and could indeed result in enhanced skeletal muscle mitochondrial oxidative function and biogenesis, at least in the early stages of weight gain [38,39]. Elevated fat substrates could also per se directly enhance mitochondrial ATP production [29], and excess fatty acids may also indeed characterize obesity independently of dietary intake [37,53]. Lower mitochondrial superoxide production is consistent with higher mitochondrial quality in obese compared to lean CHF, as indicated by higher ATP production rates, and these improvements could also potentially reflect improved mitochondrial dynamics (fusion and fission processes) [18,54]. Although obesity has been reported to lead to enhanced mitochondrial fission that may contribute to its metabolic complications, previous studies also suggested that fatty acids may indeed reduce cardiac muscle mitochondrial fragmentation [55,56,57]. Differential effects of CHF and obesity per se on different sources of superoxide are an additional intriguing finding of our study. At variance with the obese CHF group, altered tissue redox state in lean CHF animals appears to primarily involve enhanced superoxide production at mitochondrial level, with additional increments in NOS superoxide production. In obese CHF, higher total muscle superoxide production did not involve mitochondria, while suggesting a specific role for xanthine oxidase. The less pronounced impact of differential ROS sources on tissue redox state as reflected by GSSH-GSH ratio in obese CHF could be due at least in part to lower absolute ROS amounts generated in vivo at the xanthine oxidase than mitochondrial level [58], to higher activation of antioxidant defenses [59], or both. Future studies should be designed to directly address this question.

Improved tissue redox state likely causally contributed to improve pro-inflammatory cytokine patterns as well as insulin signaling activation [27,28,60,61]. Interestingly, our data show that the improved muscle cytokine profile observed in obese CHF mice is characterized by higher expression of anti-inflammatory IL-10 rather than to lower pro-inflammatory Il-1β and TNFα. This finding is in agreement with previous studies reporting beneficial effects of increased IL-10 skeletal muscle levels on insulin resistance in obese mice [62]. Also consistent with the current results in obese CHF, early paradoxical increments in insulin signaling protein activation have been notably reported in experimental high-fat diet-induced obesity models, even in the presence of elevated circulating blood glucose and systemic insulin resistance [40,41,42]. Mediators downstream of mTOR including P70S6K and PRAS40 are directly involved in activating skeletal muscle protein synthesis [63,64], and their activating phosphorylation was enhanced in obese CHF mice even beyond levels observed in non-CHF animals. This pattern is likely to directly contribute to higher skeletal muscle mass in obese CHF, and further studies should be designed to identify and characterize the molecular mechanisms of this effect. It should also be pointed out that obese animals indeed showed hyperglycemia compared to both sham and CHF lean groups, thereby indicating that lack of gastrocnemius mass loss and muscle metabolic derangements did not completely prevent systemic metabolic alterations that may characterize the obesity phenotype. Hyperglycemia in obese CHF animals could have resulted from impaired insulin action in non-skeletal muscle tissues including liver and adipose tissue, or from differential metabolic alterations in different skeletal muscle groups. Overall, moderately elevated blood glucose also further demonstrates that the obese phenotype was fully developed in the obese CHF group even after a period of pair-feeding, further supporting the potential clinical and pathophysiological relevance of the current results.

Higher gastrocnemius mass in obese CHF animals was observed both in absolute terms and relative to body weight. Higher muscle mass is notably important for its potential translational implications, since loss of skeletal muscle is an independent negative prognostic factor in CHF as well as in other chronic disease conditions [65,66,67]. As discussed above, anti-catabolic effects of improved redox state, cytokine patterns and insulin signaling are likely to directly contribute to higher gastrocnemius mass in the current obese CHF model [27,28,60,61]. It should also be noted that higher skeletal muscle mass has been demonstrated in healthy human cohorts with increasing body mass index [43,68], likely reflecting high nutrient availability and elevated muscle workload from higher body weight. Muscle weight could not be measured before induction of heart failure in the current model, and it is likely that higher muscle mass preceded the onset of catabolic conditions in obese CHF animals. The above combined observations support the hypothesis that obesity-associated higher muscle mass may be preserved after the onset of chronic disease by less catabolic patterns of tissue energy metabolism, oxidative stress, inflammation and insulin signaling. 

We designed the study to investigate the impact of obesity per se, by pair-feeding obese CHF animals to the same nutrient intake as lean counterparts. This approach is clinically pertinent, since major clinical guidelines recommend dietary control of cardiovascular risk factors following cardiovascular events through a reduction in saturated fat calories [69,70]. The selected dietary approach notably allowed to avoid the potential confounding metabolic effects of persistent high-fat feeding, since high-fat feeding per se and chronic high-fat diet-induced obesity have been reported to negatively affect mitochondrial function, mitochondrial dynamics, ROS generation and tissue inflammation [28,29,37]. It is indeed well possible that continuing high-fat feeding after CHF induction could result in independent derangement of these metabolic pathways with loss of potentially beneficial impact of obesity per se, or that further increments in body weight and body fat independently lead to deleterious muscle metabolic alterations. Our overall results suggest that clinical recommendations to limit dietary calories and fat in obese patients with CHF could also extend their potential benefits to preservation of skeletal muscle mass. Future studies should investigate further optimization of nutritional treatment for maintenance of skeletal muscle mass in obese CHF, with particular regard to optimal calorie-protein ratio and additional muscle-anabolic effect of selective elevation of dietary protein [71]. 

Some limitations of the study should finally be discussed. First, we did not include an obese sham non-CHF group. However, the current study was not designed to directly assess potential specific alterations induced by obesity in healthy animals and to compare the extent of the obesity impact in healthy and CHF animals, but rather to investigate whether muscle metabolic parameters and different muscle masses are involved in, and might therefore contribute to explaining, the reported clinical advantages in obese compared to lean patients in the context of CHF. Second, we did not measure muscle function parameters. While skeletal muscle mass and function are generally related, changes in one parameter following specific interventions do not necessarily predict the other, since function may be affected by altered neuromuscular signaling and muscle contractile quality [72,73]. We therefore cannot assume proportional changes occurring in muscle function and mass in the current model; skeletal muscle mass preservation has however independent clinical relevance, based on previous demonstrations that muscle mass loss per se is associated with negative outcomes in CHF patients [65] as well as other disease conditions [66,67]. 

In conclusion, our findings demonstrate that CHF negatively affects skeletal muscle mitochondrial function in lean rodents with lower ATP and higher mitochondrial ROS production, associated with tissue pro-inflammatory cytokine profile, lower insulin signaling and muscle mass loss. Following the onset of CHF, obesity per se is associated with higher skeletal muscle mass and tissue ATP production, lower mitochondrial ROS production, improved redox state, cytokine profile and insulin signaling. The current results identify paradoxical and potentially favorable obesity-associated metabolic patterns that could contribute to reported obesity-induced survival advantage in CHF.

## Figures and Tables

**Table 1:** Animal characteristics. Impact of previous 12-weeks diet-induced obesity in a mouse model after myocardial infarction-induced chronic heart failure and 8-week pair-feeding (ObCHF) on body weight, caloric intake, fat pads mass and plasma glucose levels compared to chronic heart failure (CHF) and previously lean (LCHF) and to lean sham operated animals (Sham). Data timings refer to study start (week 0: T0), surgical CHF induction (week 12: T12) and sacrifice (week 20: T20). *p* < 0.05 among groups not sharing a letter. * *p* < 0.05 vs. T0; mean ± standard error of mean (SEM), *n* = 8/group.

		Sham	LCHF	ObCHF
Body Weight [g]	T0	42.0 ± 1.5 ^a^	42.4 ± 0.9 ^a^	41.9 ± 1.6 ^a^
Body Weight [g]	T12	44.1 ± 1.2 ^a^	43.1 ± 1.3 ^a^	53.0 ± 0.9 ^b,^*
Body Weight [g]	T20	46.9 ± 1.3 ^a^^,*^	43.9 ± 1.1 ^b^	53.6 ± 1.4 ^c,^*
Average daily caloric intake (kcal/d)	T0-T12	7.7 ± 0.6 ^a^	7.8 ± 0.3 ^a^	25.5 ± 4.1 ^b^
Average daily caloric intake (kcal/d)	T13-T20	7.7 ± 0.9 ^a^	8.1 ± 0.4 ^a^	8.9 ± 2.1 ^a,^*
Left Retroperitoneal Fat Pad mass [mg]	T20	294 ± 70 ^a^	249 ± 58 ^b^	493 ± 72 ^c^
Left Epididimal Fat Pad mass [mg]	T20	1031 ± 161 ^a^	675 ± 162 ^b^	1351 ± 126 ^c^
Plasma Glucose [mg/dl]	T20	131 ± 4 ^a^	140 ± 3 ^a^	155 ± 3 ^b^
